# Chemical Composition and Antigerminative Activity of the Essential Oils from Five *Salvia* Species

**DOI:** 10.3390/molecules15020735

**Published:** 2010-02-01

**Authors:** Laura De Martino, Graziana Roscigno, Emilia Mancini, Enrica De Falco, Vincenzo De Feo

**Affiliations:** Dipartimento di Scienze Farmaceutiche, Università degli Studi di Salerno, Via Ponte Don Melillo, 84084 Fisciano, Salerno, Italy; E-Mails: ldemartino@unisa.it (L.D.M.); groscigno@unisa.it (G.R.); emancini@unisa.it (E.M.); edefalco@unisa.it (E.D.F.)

**Keywords:** *Salvia africana*, *Salvia elegans*, *Salvia greggii*, *Salvia mellifera*, *Salvia munzii*, essential oil, germination, radical elongation

## Abstract

The chemical composition of the essential oils of *Salvia africana* L., *Salvia elegans* Vahl, *Salvia greggii* A. Gray, *Salvia mellifera* Green and *Salvia munzii* Epling, cultivated in Eboli (Salerno, Southern Italy), was studied by means of GC and GC-MS analyses. In all, 88 compounds were identified, 54 for *S. africana*, accounting for 95.4% of the total oil, 55 for *S. elegans* (92.9%), 50 for *S. greggii* (96.9%), 54 for *S. mellifera* (90.4%) and 47 for *S. munzii* (97.5%), respectively. In *S. africana*, the amount of monoterpenoids and sesquiterpenoids is very similar. For other species, the monoterpenoid percentage is greater than the amount of sesquiterpenoids. The oils of *S. elegans*, *S. greggii* and *S. munzii* were active inhibitors of germination and radical elongation of *Raphanus sativus* L. and *Lepidium sativum* L.

## 1. Introduction

Allelopathy is an expression of the general chemical interaction among plants: a large number of plants possess both inhibitory and stimulatory effects on the growth of neighbouring or successional plants by releasing chemicals into the soil [1,2,3]. The study of plant compounds, which inhibit or stimulate the germination and the development of other species, is important for understanding the mechanisms of the ecological interaction. Our research group is studying the possible allelopathic effects of medicinal and aromatic plants [4,5,6,7] that, being rich in active principles, are considered an important source of potential allelochemicals.

The genus *Salvia* (Lamiaceae: subfamily Nepetoideae, tribe Mentheae) is a cosmopolitan assemblage of nearly 1,000 species showing a remarkable diversity in growth forms, secondary compounds, floral morphology and pollination biology. “*Salvia* phenomenon” is one of the best known and well-studied examples of allelopathy [8]: Muller and co-workers showed the potent potential allelopathic of *Salvia leucophylla* Greene and *S. apiana* Jeps. [8,9,10,11,12].

*Salvia africana* L. is an aromatic, hardy shrub up to 2 m in height, originating from Africa. There are no literature data about the essential oil of *Salvia africana* L. *Salvia elegans* Vahl is a perennial shrub native to Mexico, commonly known as “pineapple sage” and “pineapple-scented sage” in English, and “mirto”, “flor del cerro”, “limoncillo” and “perritos rojos”, in Spanish. The volatiles of pineapple-scented sage were analyzed for the first time by Makino and coworkers [13]. *S. elegans* is widely used in Mexican traditional medicine for alleviate Central Nervous System ailments [14]; Herrera-Ruiz and co-workers reported this species as a possible source for isolating new anxiolytic and antidepressant substances [15]. Moreover, Wake and coworkers studied this species for its cholinergic activity [16]. *Salvia greggii* A. Gray, “autumn sage” or “autumn salvia”, a biennial plant originating from both Mexico and the Texas, is a semi-woody species that has a showy display of brilliant red, pink, white or orange flowers from spring until the first frost in fall [17]. Only few phytochemical reports are available about *S. greggii*, in particular concerning its diterpenoid compounds [18,19,20]. *Salvia mellifera* Greene grows abundantly in California at heights below 2,000 feet and it undergoes various hybridizations with other species of the same genus [21]. *S. mellifera* is a dominant species in much of the California coastal scrub sage and bordering chaparral. Several studies reported the chemical composition of this species [22,23,24] and the biological activity of its volatile terpenes [8]. In northernmost Baja California as the coastal sage scrub becomes increasingly xeric*, S. mellifera* is replaced by *S. munzii* [22]. The chemical composition of the essential oil of *S. munzii* was studied before [22], showing the presence of camphor, 1,8-cineole and limonene, as main constituents.

In continuation of our studies on the possible phytotoxic activity of essential oils from Mediterranean plants [7,25,26], we studied the chemical composition of the essential oils from these *Salvia* species and their possible *in vitro* effects against germination and initial radical elongation of *Raphanus sativus* L. (radish) and *Lepidium sativum* L. (garden cress).

## 2. Results and Discussion

### 2.1. Chemical composition of the essential oils

Table 1 shows the chemical composition of the five *Salvia* oils; compounds are listed according to their linear retention indices (LRIs) on a HP 5MS column. In all, 88 compounds were identified, 54 for *S. africana*, accounting for 95.4% of the total oil, 55 for *S. elegans* (92.9%), 50 for *S. greggii* (96.9%), 54 for *S. mellifera* (90.4%) and 47 for *S. munzii* (97.5%), respectively.

**Table 1:** Essential oil composition of *Salvia africana*, *Salvia elegans*, *Salvia greggii*, *Salvia mellifera* and *Salvia munzii*.

Compound	Ri^a^		Ri^b^	*Salvia africana*	*Salvia elegans*	*Salvia greggii*	*Salvia mellifera*	*Salvia munzii*	Identi-fication^c^	Classi-fication ^d^	
Tricyclene	925		1013	0.7	0.2		0.2	0.3	1, 2	M	
α-Thujene	928		1035	0.2			0.3		1, 2	M	
α-Pinene	938		1032				9.2		1, 2, 3	M	
Camphene	953		1076		0.2		0.6		1, 2, 3	M	
Sabinene	973		1132	0.4	0.3		0.6		1, 2	M	
β-Pinene	980		1118	0.8	0.7	0.2			1, 2, 3	M	
Myrcene	993		1174				2.0		1, 2	M	
α-Phellandrene	1005		1150				0.1		1, 2, 3	M	
δ-3-Carene	1008		1160	1.6					1, 2, 3	M	
α-Terpinene	1013		1189	1.7	0.1		0.8	0.1	1, 2, 3	M	
*o*-Cymene	1020		1187		0.1	0.2	0.5	0.1	1, 2, 3	M	
*p*-Cymene	1025		1280	21.2					1, 2, 3	M	
β-Phellandrene	1029		1218		0.4				1, 2, 3	M	
Limonene	1030		1203	0.4	1.1	0.4	2.2	1.4	1, 2, 3	M	
1,8-Cineole	1034		1213	0.2	0.4	0.2	39.8	0.2	1, 2, 3	MO	
(*Z*)-β-Ocimene	1038		1243	1.1	2.2	0.1	0.4	5.7	1, 2	M	
(*E*)-β-Ocimene	1049		1262		0.1	0.1	0.2	0.2	1, 2	M	
*γ-*Terpinene	1057		1256	15.5	0.1	0.1	2.0	0.2	1, 2, 3	M	
*cis*-Sabinene hydrate	1063		1556	0.2	0.1	0.1	0.2	0.1	1, 2	MO	
*trans*-Linalool oxide	1085		1455		0.1	0.1		0.1	1, 2	MO	
*trans-* Sabinene hydrate	1093		1474	1.3			1.0		1, 2	MO	
*cis*-Thujone	1105		1430	0.2	38.7	43.4	0.2	33.3	1, 2	MO	
2-Phenyl ethyl alcool	1113		1925	0.2		3.3		2.0	1, 2, 3	MO	
*trans-*Thujone	1115		1449	0.4					1, 2	MO	
*cis*-*p* -Menth-2-en-1-ol	1128		1638	0.2	0.1	0.1			1, 2	MO	
Camphor	1145		1532	0.2	4.6	4.2	12.2	27.2	1, 2, 3	MO	
Pinocarvone	1165		1587	0.1					1, 2	MO	
Borneol	1167		1719	0.4					1, 2, 3	MO	
Terpinen -4-ol	1176		1611	1.0	0.7	0.7	2.0	0.6	1, 2, 3	MO	
*p-*Cymen-8-ol	1185		1856	0.1	0.1	0.1	0.1	0.1	1, 2	MO	
α-Terpineol	1189		1706	0.5	1.6	2.0	0.7	1.2	1, 2	MO	
Verbenone	1204		1723	0.1					1, 2	MO	
*trans-*Carveol	1217		1845	0.1			0.1		1, 2	MO	
Myrtenyl acetate	1227		1698				0.1		1, 2	MO	
Geraniol	1235		1857		6.5	3.4	0.1	4.0	1, 2	MO	
Neral	1240		1656		0.7	0.5		0.6	1, 2	MO	
Carvone	1241		1752			0.1			1, 2, 3	MO	
Geranial	1267		1712		1.0	0.5		0.5	1, 2, 3	MO	
Bornyl acetate	1284		1597	1.7	1.0	1.2	0.5	0.3	1,2	MO	
Thymol	1293		2198	0.8		1.6		1.1	1, 2, 3	P	
Carvacrol	1299		2239	0.5	0.6	0.8		0.4	1, 2, 3	P	
δ-Elemene	1335		1476		0.1	0.1		0.1	1, 2	S	
α-Cubebene	1352		1466	0.2	0.1	0.4		0.1	1, 2	S	
(*Z*)-Isoeugenol	1353		2186	0.2	0.1		0.1	0.1	1, 2	P	
Citronellyl acetate	1358		1662			0.1			1, 2	MO	
Neryl acetate	1367		2097		0.2	0.2		0.1	1, 2	MO	
Geranyl acetate	1379		1765		6.9	8.7		2.0	1, 2	MO	
β-Elemene	1387		1600		0.4	0.4		0.2	1, 2	S	
α-Gurjunene	1408		1529	0.2	0.1				1, 2	S	
β-Caryophyllene	1415		1612	0.4	0.2	0.1	0.9	0.1	1, 2	S	
Aromadendrene	1422		1628	0.4	0.1		0.1	0.1	1, 2	S	
β-Gurjunene	1431		1632	0.2		0.1	0.1		1, 2	S	
γ-Elemene	1434		1650				0.4		1, 2	S	
α-Guaiene	1437		1530	1.0	0.5		0.1	0.1	1, 2	S	
*trans-*Bergamotene	1438						0.1		1, 2	S	
α-Humulene	1455		1689	0.4	0.3		0.2	0.2	1, 2	S	
*allo*-Aromadendrene	1463		1661		0.2	0.2	0.1		1, 2	S	
γ-Gurjunene	1473		1687	0.1					1, 2	S	
Germacrene D	1477		1726	0.1	0.2	0.2	0.1	0.2	1, 2	S	
γ-Muurolene	1478		1704		0.1	0.1	0.1	0.1	1, 2	S	
*cis-*β-Guaiene	1490		1694	0.2	0.2	0.2	0.2	0.2	1,2	S	
Biciclogermacrene	1491		1756		2.5	1.7		1.1	1, 2	S	
Valencene	1495		1741	0.4	0.5	0.4	0.2	0.3	1, 2	S	
α-Selinene	1498		1744				0.4		1, 2	S	
α-Muurolene	1500		1740	0.4	1.8	2.3	0.1	1.4	1, 2	S	
β-Himachalene	1505		1706	0.4	0.1	0.1	0.3		1, 2	S	
β-Bisabolene	1510		1743				0.7		1, 2	S	
γ-Cadinene	1515		1776	2.8	1.5	1.3	0.3	1.0	1, 2	S	
Cubebol	1517		1957	0.2	0.2		0.1		1, 2	SO	
*cis*-Calamenene	1520		1839		0.1	0.1			1, 2	S	
Selina-3,7(11)-diene	1524			1.7			0.5		1, 2	S	
δ-Cadinene	1526		1773	4.6	11.5	14.0	0.9	8.9	1, 2	S	
α-Cadinene	1535		1745		0.3	0.3		0.2	1, 2	S	
Cadina-1,4-diene	1538		1799		0.1	0.1		0.1	1, 2	S	
α-Calacorene	1541		1941			0.1			1, 2	S	
Germacrene B	1544		1854	0.2			1.1		1, 2	S	
Germacrene D-4-ol		1577	2069	0.5			0.5		1,2	SO	
Spathulenol	1578		2150		0.2				1, 2	SO	
Caryophyllene oxide	1580		2008	1.3			1.4		1, 2, 3	SO	
Globulol	1585		2098	0.2			1.8	0.2	1, 2	SO	
Viridiflorol	1591		2104	0.2					1, 2	SO	
β-Oplopenone	1608		2100		0.2	0.1	0.8	0.1	1, 2	SO	
1- *epi*-Cubenol	1625		2088	2.9	0.2	0.2		0.1	1, 2	SO	
τ-Cadinol	1640		2187	13.6	0.9	0.8	0.4	0.3	1, 2	SO	
τ-Muurolol	1642		2209		1.4	1.1	0.4	0.5	1, 2	SO	
α-Cadinol	1649		2255				1.9			SO	
α-Eudesmol	1652		2250	10.7					1, 2	SO	
TOTAL				95.4	92.9	96.9	90.4	97.5			
Monoterpene hydrocarbons				43.6	5.5	1.1	19.1	8			
Oxygenated Monoterpenes				7.1	62.7	68.9	57	72.3			
Phenolic compounds				1.6	0.7	2.4	0.1	1.6			
Sesquiterpene hydrocarbons				13.6	20.9	22.3	6.9	14.4			
Oxygenated Sesquiterpenes				29.6	3.1	2.2	7.3	1.2			

^a^ Kovats retention index on HP-5 MS column; ^b^ Kovats retention index on HP Innowax; ^c^ 1 = Kovats retention index, 2 = mass spectrum, 3 = coinjection with authentic compound; ^d^ M = Monoterpene hydrocarbons, MO = Oxygenated Monoterpenes, P = Phenolic compounds, S = Sesquiterpene hydrocarbons, SO = Oxygenated Sesquiterpenes.

In *S. africana* the monoterpenes and sesquiterpenes were almost in a similar percentage, amounting to 50.6% and 43.2%, respectively. The main compounds are *p*-cymene (21.2%), γ-terpinene (15.5%), both monoterpenes, τ-cadinol (13.6%) and α-eudesmol (10.7%), oxygenated sesquiterpenes. Other sesquiterpene compounds, in less amount, are δ-cadinene (4.6%) and γ-cadinene (2.8%).

In the oil from *S. elegans*, the monoterpenes amounted to 68.2% and consisted mainly of oxygenated compounds (62.7%); on the other hand, the total sesquiterpenes were 24.0% (20.9% sesquiterpene hydrocarbons and 3.1% of oxygenated sesquiterpenes) of the total oil. *cis*-Thujone (38.7%) and geranyl acetate (6.9%) were the most abundant among oxygenated monoterpenes, while geraniol (6.5%) and camphor (4.6%) were present in less amount. The most abundant sesquiterpene hydrocarbons were δ-cadinene (11.5%), bicyclogermacrene (2.5%) and α-muurolene (1.8%). The volatiles of pineapple-scented sage were analyzed for the first time by Makino and coworkers [13] and 28 constituents were identified: among them, mono- and sesquiterpenoids such as linalool, β-caryophyllene, germacrene D and spathulenol were the predominant components.

In the oil from *S. greggii*, the monoterpene fraction amounted to 70.0% of the total oil, while sesquiterpenes accounted for only 24.5%. In the monoterpene fraction, oxygenated monoterpenes represent a great amount, accounting for 68.9%. The main components were *cis*-thujone (43.4%) and geranyl acetate (8.7%), while camphor (4.2%) and geraniol (3.4%) were in less amount. δ-Cadinene (14.0%), α-muurolene (2.3%) and biciclogermacrene (1.7%) were the most abundant sesquiterpene hydrocarbons.

In the *S. mellifera* oil, the monoterpene fraction amounted to 76.1%, while the sesquiterpene one was 14.2%. Also in this case, the monoterpene fraction was mainly represented by oxygenated compounds (57.0%), with great prevalence of 1,8-cineole (39.8%) and camphor (12.2%). α-Pinene (9.2%) was the major component of non-oxygenated monoterpenes. Neisess and coworkers [22] reported camphor as one of the most abundant compounds in *S. mellifera* and *S. munzii* oils; our data confirmed their results.

In *S. munzii*, the monoterpene fraction amounted to 80.3% of the total oil, while sesquiterpenes represented only 15.6%: the main compounds are *cis*-thujone (33.3%) and camphor (27.2%), with δ-cadinene (8.9%) and geraniol (4%) in appreciable amounts. In literature, Neisses and coworkers [22] reported the essential oil composition of this species and they showed that camphor is one of the major compound of the oil: once more, our data confirmed their results.

### 2.2. Biological activity

The five essential oils were evaluated for their phytotoxic activity against germination (Table 2) and initial radical elongation (Table 3) of radish (*Raphanus sativus*) and garden cress (*Lepidium sativum*), two species usually utilized in biological assays [7]. The oils affected the germination and the radical elongation of two seeds in a distinct way. The germination of radish appeared sensitive to *Salvia greggii* oil, at the highest dose (1.25 μg/mL) used. The germination of garden cress was completely inhibited by *S. elegans*, *S. greggii* and *S. munzii* oils, at the highest doses (1.25 μg/mL, 0.625 μg/mL) used. The essential oil of *S. elegans*, at the almost all doses tested, inhibited significantly the radical elongation of both radish and garden cress. Also *S. greggii* and *S. munzii* oils inhibited, in a significative way, the radical elongation as of radish as of garden cress; on the other hand, *S. mellifera* oil inhibited, in a significative way, the radical elongation of radish but not of garden cress, while *S. africana* oil was inactive towards both seeds.

**Table 2:** Biological activity of essential oils of *Salvia africana, Salvia elegans, Salvia greggii*, *Salvia mellifera* and *Salvia munzii*, against germination of *Raphanus sativus* and *Lepidium sativum*, 120 h after sowing. Results are the mean of three experiments ± SD.

Raphanus sativus Germinated seeds ± SD					
**Doses**	***Salvia africana***	***Salvia elegans***	***Salvia greggii***	***Salvia mellifera***	***Salvia munzii***
Control	9.3 ± 1.1	9.3 ± 1.1	9.3 ± 1.1	9.3 ± 1.1	9.3 ± 1.1
0.06 μg/mL	9.7 ± 0.6	7.7 ± 2.0	7.3 ± 1.5	10 ± 0	9.7 ± 0.6
0.125 μg/mL	9.0 ± 1.0	8.7 ± 1.5	8.0 ± 1.7	10 ± 0	10 ± 0
0.25 μg/mL	8.7 ± 1.5	7.6 ± 0.6	8.7 ± 1.5	8.7 ± 0.6	9.0 ± 1.0
0.625 μg/mL	9.7 ± 0.6	7.6 ± 0.6	7.6 ± 0.6	8.7 ± 1.5	9.0 ± 1.0
1.25 μg/mL	8.7 ± 1.1	7.6 ± 0.6	6.3 ± 0.6	8.3 ± 1.5	8.7 ± 1.1
**Lepidium sativum** Germinated seeds ± SD					
**Doses**	***Salvia africana***	***Salvia elegans***	***Salvia greggii***	***Salvia mellifera***	***Salvia munzii***
Control	9.3 ± 0.6	9.3 ± 0.6	9.3 ± 0.6	9.3 ± 0.6	9.3 ± 0.6
0.06 μg/mL	9.7 ± 0.6	8.3 ± 1.5	9.3 ± 1.1	8.7 ± 0.6	9.7 ± 0.6
0.125 μg/mL	9.7 ± 0.6	8.7 ± 1.5	8.0 ± 1.7	8.7 ± 0.6	9.0 ± 1.0
0.25 μg/mL	10 ± 0	6.0 ± 1.0*	7.0 ± 1.0	7.0 ± 1.0	10 ± 0
0.625 μg/mL	10 ± 0	0 ± 0	1 ± 0	6.0 ± 1.0^*^	0 ± 0
1.25 μg/mL	9.0 ± 1.0	0 ± 0	0 ± 0	6.3 ± 0.6	0 ± 0

Note:p < 0.05 *vs.* control.

**Table 3:** Biological activity of essential oils of *Salvia africana*, *Salvia elegans*, *Salvia greggii*, *Salvia mellifera* and *Salvia munzii* against radical elongation of *Raphanus sativus* and *Lepidium sativum*, 120 h after sowing. Data are expressed in cm. Results are the mean of three experiments ± SD.

Raphanus sativus Radical elongation ± S.D					
**Doses**	***Salvia africana***	***Salvia elegans***	***Salvia greggii***	***Salvia mellifera***	***Salvia munzii***
Control	3.4 ± 2.0	3.4 ± 2.0	3.4 ± 2.0	3.4 ± 2.0	3.4 ± 2.0
0.06 μg/mL	2.6 ± 1.0	2.7 ± 1.3	3.4 ± 1.9	2.4 ± 1.1*	2.1 ± 1.3**
0.125 μg/mL	3.2 ± 1.6	2.1 ± 0.9**	1.9 ± 1.3	2.6 ± 1.4	2.3 ± 0.8**
0.25 μg/mL	2.5 ± 1.5	1.9 ± 1.1**	2.7 ± 1.6	2.9 ± 1.7	1.9 ± 1.1**
0.625 μg/mL	3.1 ± 2.0	1.2 ± 0.9***	2.2 ± 1.2*	2.2 ± 0.9**	2.1 ± 1.2**
1.25 μg/mL	2.5 ± 1.4	1.4 ± 0.6***	1.3 ± 0.5***	1.9 ± 1.4**	1.2 ± 0.7***
**Lepidium sativum** Radical elongation ± S.D					
**Doses**	***Salvia africana***	***Salvia elegans***	***Salvia greggii***	***Salvia mellifera***	***Salvia munzii***
Control	2.5 ± 0.9	2.5 ± 0.9	2.5 ± 0.9	2.5 ± 0.9	2.5 ± 0.9
0.06 μg/mL	4.1 ± 2.4**	1.7 ± 0.8**	2.4 ± 0.9	2.3 ± 0.7	2.3 ± 0.7
0.125 μg/mL	2.6 ± 0.8	1.1 ± 0.8***	2.9 ± 1.5	3.3 ± 1.9	2.1 ± 0.9
0.25 μg/mL	2.4 ± 0.9	0.8 ± 0.4***	2.5 ± 0.9	2.4 ± 1.8	0.8 ± 0.6***
0.625 μg/mL	2.6 ± 0.9	0.0 ± 0.0***	2.5***	2.3 ± 0.9	0.0 ± 0.0***
1.25 μg/mL	2.6 ± 1.1	0.0 ± 0.0***	0.0 ± 0.0***	2.6 ± 1.5	0.0 ± 0.0***

Note: * p < 0.05; ** p < 0.01; *** p < 0.001 *vs.* control.

The difference in biological activity of the oils could be attributed to their different chemical composition: in *S. africana* oil, there is a minor amount of oxygenated terpenoids, reported as germination and seedling growth inhibitors [27], in comparison with the other four oils: in fact, this species of *Salvia* is the less active. On the other hand, the oils of *S. elegans, S. greggii* and *S. munzii*, that have a similar chemical composition, with *cis*-thujone, camphor and 1,8-cineole as main components, possess a comparable antigerminative activity. These compounds are known as potent inhibitors of seed germination [8]. Moreover, Pinto and coworkers [28] showed that a *Salvia officinalis* oil, containing 10.4% of *cis*-thujone and 20.5% of camphor, was very active against *Candida* sp., dermatophytes and other filamentous fungi and showed fungicidal activity: *cis*-thujone and camphor are both present in our most active oils.

Our data agree with the literature on inhibitory activity exerted by essential oils of *Salvia* species on seed germination and radical elongation and, in general, on vegetation. Muller [12] reports a dramatic example of zones free of annual herbs, influenced by terpenoids, in the areas surrounding patches of *Salvia leucophylla*. Several authors [29,30] studied the mechanism of monoterpene volatilization in *S. mellifera*. Volatile monoterpenoids, emanating from leaves of this species are responsible for anatomical and physiological changes occurring in herb seedlings which were exposed to vapours [31]. Camphor and 1,8-cineole, the main components of the oil of *Salvia leucophylla,* are potent inhibitors of oxygen uptake by mitochondrial suspensions [12]. Moreover, we reported recently the phytotoxic activity of other two species of *Salvia* [26].

Although the mode of inhibitory action of essential oils against germination still remains unclear, several papers reported that volatile oils and monoterpenoids inhibit cell division and induce structural breaks and decomposition in roots [32,33,34,35]. Both monoterpenoids and sesquiterpenoids appear to be involved in these allelopathic effects. Some monoterpenoids are potent inhibitors of seed germination and radical elongation. These include cineoles, as 1,4- and 1,8-cineole [32], citronellol, linalool [35,36], α-pinene [34,37], and limonene [37]. Recently, researchers reviewed the role of sesquiterpenoid compounds: chemicals as β-maaliene, α-isocomene, β-isocomene, δ-cadinene, 5-hydroxy-calamenene, and 5-methoxycalamenene were shown to inhibit the seedling growth of associated native vegetation, and thus possibly help in successful invasion in the introduced sites [38].

## 3. Experimental

### 3.1. Plant material

Aerial parts of *Salvia africana* L., *Salvia elegans* Vahl, *Salvia greggii* A. Gray, *Salvia mellifera* Green and *Salvia munzii* Epling were gathered at the full flowering stage from plants cultivated in the garden of the Improsta Experimental Station at Eboli, (Salerno), in July 2009. The seeds of the plants were purchased at “Vivaio Granburrone”, Nocera Umbra (Perugia, Italy). Plants were identified by Prof. V. De Feo. Voucher specimens of each plant were deposited in the Herbarium of the Medical Botany Chair at the Salerno University. The specimens are labelled as follows: *S. africana* (DF 2009/345), *S. elegans* (DF 2009/346), *S. greggii* (DF 2009/347) *S. mellifera* (DF 2009/348), *S. munzii* (DF 2009/349).

### 3.2. Isolation of the volatile components

Fifteen grams of each air-dried sample of *Salvia* species were ground in a Waring blender and then subjected to hydrodistillation for 3 h according to the standard procedure described in the European Pharmacopoeia [39]. The oils were solubilised in *n*-hexane, filtered over anhydrous sodium sulphate and stored under N_2_ at +4 °C in the dark until tested and analyzed. The dry materials gave yellow-reddish oils in a yield of 0.37% (v/w) for *S. africana*, of 0.55 (v/w) for *S. elegans*, of 0.70% (v/w) for *S. greggii*, 0.68% (v/w) for *S. mellifera* and 0.80% (v/w) for *S. munzii*.

### 3.3. Gas chromatography

Analytical gas chromatography was carried out on a Perkin-Elmer Sigma-115 gas chromatograph equipped with a FID and a data handling processor. The separation was achieved using a HP-5 MS fused-silica capillary column (30 m × 0.25 mm i.d., 0.25 μm film thickness). Column temperature: 40 °C, with 5 min initial hold, and then to 270 °C at 2 °C/min, 270 °C (20 min); injection mode splitless (1 μL of a 1:1,000 *n*-pentane solution). Injector and detector temperatures were 250 °C and 290 °C, respectively. Analysis was also run by using a fused silica HP Innowax polyethylenglycol capillary column (50 m × 0.20 mm i.d., 0.25 μm film thickness). In both cases, helium was used as carrier gas (1.0 mL/min).

### 3.4. Gas chromatography–Mass spectrometry

Analysis was performed on an Agilent 6850 Ser. II apparatus, fitted with a fused silica DB-5 capillary column (30 m × 0.25 mm i.d., 0.33 μm film thickness), coupled to an Agilent Mass Selective Detector MSD 5973; ionization energy voltage 70 eV; electron multiplier voltage energy 2,000 V. Mass spectra were scanned in the range 40–500 amu, scan time 5 scans/s. Gas chromatographic conditions were as reported in the previous paragraph; transfer line temperature, 295 °C.

### 3.5. Identification of components

Most constituents were identified by gas chromatography by comparison of their Kovats retention indices (Ri) with either those of the literature [40,41] or with those of authentic compounds available in our laboratories. The Kovats retention indices were determined in relation to a homologous series of *n*-alkanes (C_8_–C_28_) under the same operating conditions. Further identification was made by comparison of their mass spectra on both columns with either those stored in NIST 02 and Wiley 275 libraries or with mass spectra from the literature [40,42] and a home made library. Components relative concentrations were obtained by peak area normalization. No response factors were calculated.

### 3.6. Biological assay

A bioassay based on germination and subsequent radical growth was used to study the phytotoxic effects of the essential oils of *S. africana*, *S. elegans*, *S. greggii*, *S. mellifera* and *S. munzii* on seeds of *Raphanus sativus* L. cv. “Saxa” (radish), and *Lepidium sativum* L. (garden cress). The seeds were purchased from Blumen srl, Piacenza, Italy. The seeds were surface sterilized in 95% ethanol for 15 s and sown in Petri dishes (Ø = 90 mm), containing five layers of Whatman filter paper, impregnated with distilled water (7 mL, control) or tested solution of the essential oil (7 mL), at the different assayed doses. The germination conditions were 20 ± 1 °C, with natural photoperiod. The essential oils, in water–acetone mixture (99.5:0.5), were assayed at the doses of 1.25, 0.625, 0.25, 0.125 and 0.062 μg/mL. Controls performed with water–acetone mixture alone showed no appreciable differences in comparison with controls in water alone. Seed germination was observed directly in Petri dishes, each 24 h. Seed was considered germinated when the protrusion of the radical became evident [43]. After 120 h (on the fifth day), the effects on radical elongation were measured in cm. Each determination was repeated three times, using Petri dishes containing 10 seeds each. Data are expressed as the mean ± SD of both germination and radical elongation. The Student’s t test of independence was applied [44].

## 4. Conclusions

Aromatic plants are regarded as a primary source of potential allelochemicals and interact with their environment. Muller and coworkers demonstrated [8,9,10,11,12] that *Salvia* species produce volatile growth inhibitors, particularly oxygenated monoterpenoids. These findings were subsequently confirmed by other papers [13,29]. Our *in vitro* experiments on the essential oils from *Salvia* species on germination and initial radical elongation of radish and garden cress, show that the essential oils of *S. elegans* and *S. munzii* were the most active inhibitors *,* whereas *S. africana* oil didn’t show such activity. The phytotoxic activity of *S. elegans* and *S. munzii* was probably due to the presence of a substantial amount of oxygenated terpenoids, in particular of *cis*-thujone, 1,8-cineole and camphor. Our *in vitro* studies can contribute to explain the importance of volatile compounds as chemical mediators in biochemical interactions among higher plants and could suggest models for lead compounds in the development of new pesticides [45].

## References

[1] Harborne J.B. (1988). Introduction to Ecological Biochemistry.

[2] Inderjit (1996). Plant phenolics in allelopathy. Bot. Rev..

[3] Seigler D.S. (1996). Chemistry and mechanisms of allelopathic interactions. Agron.J..

[4] Aliotta G., Cafiero G., De Feo V., Palumbo D., Strumia S. (1996). Infusion of rue for control of purslane weed: Biological and chemical aspects. AllelopathyJ..

[5] De Feo V., De Simone F., Senatore F. (2002). Potential allelochemicals from essential oil of *Ruta graveolens*. Phytochemisty.

[6] Rolim de Almeida L.F., Sannomiya M., De Feo V., Rodrigues C.M., Delachiave M.E., Campaner dos Santos L., Hiruma-Lima C., Vilegas W. (2007). Allelopathic effects of extracts and amenthoflavone from *Birsonyma crassa* (Malpighiaceae). J. Plant Interact..

[7] Mancini E., Arnold N.A., De Feo V., Formisano C., Rigano D., Piozzi F., Senatore F. (2009). Phytotoxic effects of essential oils of *Nepeta curviflora* Boiss. and *Nepeta nuda* L. subsp. *albiflora* growing wild in Lebanon. J. Plant Interact..

[8] Muller C.H. (1965). Inhibitory terpenes volatilized from *Salvia* shrubs. Bull. Torrey Bot. Club.

[9] Muller W.H., Muller C.H. (1964). Volatile growth inhibitors produced by *Salvia* species. Bull. Torrey Bot. Club.

[10] Muller C.H., Muller W.H., Haines B.L. (1964). Volatile growth inhibitors produced by aromatic shrubs. Science.

[11] Muller W.H., Lorber P., Haley B. (1968). Volatile growth inhibitors produced by *Salvia leucophylla*: Effect on seedling growth and respiration. Bull. Torrey Bot. Club.

[12] Muller W.H., Lorber P., Haley B., Johnson K. (1969). Volatile growth inhibitors produced by * Salvia leucophylla*: Effects on oxygen uptake by mitocondrial suspensios. Bull. Torrey Bot. Club.

[13] Makino T., Ohno T., Iwbuchi H. (1996). Aroma components of pineapple sage (*Salvia elegans* Vahl). Foods Food Ingred. J. Jpn..

[14] Mora S., Millán R., Lungenstrass H., Díaz-Véliz G., Morán J.A., Herrera-Ruiz M., Tortoriello J. (2006). The hydroalcoholic extract of *Salvia elegans* induces anxiolytic- and antidepressant-like effects in rats. J. Ethnopharmacol..

[15] Herrera-Ruiz M., Garcia-Beltran Y., Mora S., Diaz-Veliz G., Viana Glauce S.B., Tortoriello J., Ramirez G. (2006). Antidepressant and anxiolytic effects of hydroalcoholic extract from * Salvia elegans*. J. Ethnopharmacol..

[16] Wake G., Court J., Pickering A., Lewis R., Wilkins R., Perry E. (2000). CNS acetylcholine receptor activity in European medicinal plants traditionally used to improve failing memory. J. Ethnopharmacol..

[17] Frett J.F. (1987). Influence of nutrient salts, auxins and cytokinins on the *in vitro* growth of *Salvia greggii*. Plant Cell Tiss. Org..

[18] Bruno M., Savona G., Fernandez-Gadea F., Rodriguez B. (1986). Diterpenoids from *Salvia greggii*. Phytochem..

[19] Kawahara N., Inoue M., Kawai K., Sekita S., Satake M., Goda Y. (2003). Diterpenoid from *Salvia greggii*. Phytochem..

[20] Kawahara N., Tamura T., Inoue M., Hosoe T., Kawai K., Sekita S., Satake M., Goda Y. (2004). Diterpenoid glucosides from *Salvia greggii*. Phytochem..

[21] Moujir L., Gutierrez-Navarro A.M., San Andres L., Javier G.L. (1996). Bioactive diterpenoids isolated from *Salvia mellifera*. Phytother. Res..

[22] Neisess K.R., Scora R.W., Kumamoto J. (1987). Volatile leaf oils of California Salvias. J. Nat. Prod..

[23] Luis J.G., Andres L.S. (1999). An eremophylane-type sesquiterpene and diterpenes from roots of *Salvia mellifera*. Nat. Prod. Lett..

[24] Marrero J.G., San Andres L., Luis J.G. (2005). Quinone derivatives by chemical transformations of 16-hydroxycarnosol from *Salvia* species. Chem. Pharm. Bull..

[25] Arminante F., De Falco E., De Feo V., De Martino L., Mancini E., Quaranta E. (2006). Allelopathic activity of essential oils from Mediterranean Lamiaceae. Acta Hort..

[26] Mancini E., Arnold N.A., De Martino L., De Feo V., Rigano D., Senatore F. (2009). Chemical composition and phytotoxic effects of essential oils of *Salvia* hierosolymitana Boiss. and *Salvia multicaulis* Vahl. var. *simplicifolia* Boiss. growing wild in Lebanon. Molecules.

[27] Kordali S., Cakir A., Sutay S. (2007). Inhibitory effects of monoterpenes on seed germination and seedling growth. Z. Naturforsch. C..

[28] Pinto E., Salgueiro L.R., Cavaleiro C., Palmeira A., Goncalves M.J. (2007). *In vitro* susceptibility of some species of yeasts and filamentous fungi to essential oils of *Salvia officinalis*. Ind. Crop. Prod..

[29] Tyson B.J., Dement W.A., Mooney H.A. (1974). Volatilization of terpenes from *Salvia mellifera*. Nature.

[30] Dement W.A., Tyson B.J., Mooney H.A. (1975). Mechanism of monoterpene volatilization in *Salvia mellifera*. Phytochemisty.

[31] Lorber P., Muller W.H. (1976). Volatile growth inhibitors produced by *Salvia leucophylla*: Effects on seedling root tip ultrastructure. Am. J. Bot..

[32] Romagni J.G., Allen S.N., Dayan F.E. (2000). Allelopathic effects of volatile cineoles on two weedy plant species. J. Chem. Ecol..

[33] Nishida N., Tamotsu S., Nagata N., Saito C., Sakai A. (2005). Allelopathic effects of volatile monoterpenoids produced by *Salvia leucophylla*: Inhibition of cell proliferation and DNA synthesis in the root apical meristem of *Brassica campestris* seedlings. J. Chem. Ecol..

[34] Singh H.P., Batish D.R., Kaur S., Arora K., Kohli R.K. (2006). α-Pinene inhibits growth and induces oxidative stress in roots. Ann. Bot..

[35] Singh H.P., Batish D.R., Kaur S., Kohli R.K., Arora K. (2006). Phytotoxicity of volatile monoterpene citronellal against some weeds. Z. Naturforsch. C..

[36] Singh H.P., Batish D.R., Kaur S., Ramezani H., Kohli R.K. (2002). Comparative phytotoxicity of four monoterpenes against *Cassia occidentalis*. Ann. Appl. Biol..

[37] Abrahim D., Braguini W.L., Kelmer Bracht A.M., Ishiiwamoto E.L. (2000). Effects of four monoterpenes on germination primary root growth and mitochondrial respiration of maize. J. Chem. Ecol..

[38] Ens E.J., Bremner J.B., French K., Korth J. (2008). Identification of volatile compounds released by roots of an invasive plant, bitou bush (*Chrysanthemoides monilifera* spp. *rotundata*), and their inhibition of native seedling growth. Biol. Invasions.

[39] (2004). European Pharmacopoeia.

[40] Jennings W., Shibamoto T. (1980). Qualitative Analysis of Flavour and Fragrance Volatiles by Glass capillary Gas Chromatography.

[41] Davies N.W. (1990). Gas chromatographic retention indices of monoterpenes and sesquiterpenes on methyl silicone and Carbowax 20M phases. J. Chromatogr..

[42] Adams R.P. (2007). Identification of Essential Oil Components by Gas Chromatography/Mass Spectroscopy.

[43] Bewley D., Black M. (1985). Seeds: Physiology of Development and Germination.

[44] Sokal R.R., Rohlf F.J. (1981). Biometry.

[45] Duke S.O., Dayan F.E., Romagni J.G., Rimando A. (2000). Natural products as sources of herbicides: Current status and future trends. Weed Res..

